# Verbal Learning and Memory Function and the Influencing Factors on Breast Cancer Survivors: A Cross-Sectional Study

**DOI:** 10.31372/20190402.1036

**Published:** 2019

**Authors:** Hilman Syarif, Agung Hilman Waluyo, Yati Afiyanti, Muchtaruddin Mansyur

**Affiliations:** a Doctoral Student in the Faculty of Nursing, Universitas Indonesia, Indonesia; b Department of Medical Surgical Nursing, Faculty of Nursing, Universitas Syiah Kuala, Indonesia; c Department of Medical Surgical Nursing, Faculty of Nursing, Universitas Indonesia, Indonesia; d Department of Maternity Nursing, Faculty of Nursing, Universitas Indonesia, Indonesia; e Faculty of Medicine, Universitas Indonesia, Indonesia

**Keywords:** chemotherapy, memory function, survivors, verbal learning function

## Abstract

Impairment of cognitive function is a common complaint by post-chemotherapy breast cancer survivors, specifically impairment of verbal learning and memory. The objective of this study was to identify the association between age, duration of education, chemotherapy type, hormone therapy usage, menopausal status, sleep quality, fatigue, stress, and hemoglobin (Hb) levels to memory and verbal learning function. This cross-sectional study consisted of 82 post-chemotherapy breast cancer survivors, 81 non-chemotherapy survivors, and 80 non-cancer female patients in two hospitals. The data were collected using the Hopkins Verbal Learning Test in Indonesian, Pittsburgh Sleep Quality Index, 10-item Perceived Stress Scale, and the Piper Fatigue Scale-12. All instruments were already adopted into Indonesian. Characteristic data were obtained from hospital reports. The mean age of the respondents was 43.06 (8.18) years, 197 (81.1%) had been educated for ≤12 years, 82 (33.7%) were post-chemotherapy survivors, 46 (18.9%) were using hormonal therapy, and 125 (51.4%) had gone through menopause. Among the remaining respondents, 91 (37.4%) were anemic, 124 (51.0%) had poor sleep quality, and 115 (47.3%) experienced moderate fatigue. Twenty-one (25.6%) of post-chemotherapy survivors had a high possibility of having dementia. The significant variables associated with memory and verbal learning function included age, stress, survivor type, chemotherapy category, sleep quality, and fatigue. The insignificant variables included the length of education, hormone therapy usage, menopausal status, and hemoglobin levels. A logistic regression analysis showed that stress was the most influential variable with an odds ratio of 1.159. It is recommended that nurses consider the significant variables when providing services to post-chemotherapy breast cancer survivors.

## Introduction

One particular problem that can affect breast cancer survivors is cognitive dysfunction, which is commonly known as chemobrain or chemo-fog (Selamat, Loh, Mackenzie, & Vardy, 2014). This phenomenon is characterized by a mental disorder that encompasses many symptoms, such as short-term memory loss, difficulty in thinking and concentrating, multitasking disorders, and other subtle cognitive changes (Argyriou, Assimakopoulos, Iconomou, Giannakopoulou, & Kalofonos, 2011; Asher & Myers, 2015). Research has shown that cognitive problems cause negative effects and sometimes dramatically affect the function, quality of life, and community integration of the patient (Argyriou et al., 2011; Janelsins, Kesler, Ahles, & Morrow, 2014; Vardy, Wefel, Ahles, Tannock, & Schagen, 2007).

Approximately 16%–75% of breast cancer survivors have experienced cognitive impairment during treatment compared with 4%–11% of healthy controls (Janelsins et al., 2011). A meta-analysis of breast cancer survivors with standard dose chemotherapy for ≥6 months showed that cognitive deficits (generally with low severity) were present in these survivors, particularly with regard to verbal limitations and visuospatial abilities (Jim et al., 2012). Cognitive decline due to chemotherapy occurs in 10%–40% of cancer survivors and in approximately 23% of female survivors treated with chemotherapy (Jansen, Cooper, Dodd, & Miaskowski, 2011). Approximately 63% of cancer survivors have been found to have problems with concentration and attention, 50% have memory problems, and 38% have problems with abstract reasoning. Approximately, 75% of respondents experienced reduced working performance, 58% had to use a compensation strategy, 50% were frustrated, and 33% experienced adverse effects on family relationships (Lai et al., 2009).

Studies have highlighted cancer survivors who have reported difficulties in job activity and in maintaining functional ability levels; these survivors require increased mental support (Wefel, Witgert, & Meyers, 2008). Other studies have identified cognitive impairments such as memory loss and attention or concentration disorders (Biglia et al., 2012). Jansen et al. (2011) mentioned problems with concentration, visuospatial skills, and motor function. The cognitive changes experienced by respondents are described as frustrating conditions (Boykoff, Moieni, & Subramanian, 2009). The chemobrain experienced by respondents caused frustration, affected self-esteem and social relationships, and caused difficulties in working performance and adaptability via compensation strategies (Von Ah, Habermann, Carpenter, & Schneider, 2013). The respondents experienced memory loss, decision-making difficulties, and speech impairment. Married women reported frustration because of their diminished role in the household (Cheung et al., 2012).

Indonesia has many hospitals that provide chemotherapy services; therefore, there are many breast cancer survivors who have received chemotherapy. Numerous studies on chemobrain has been done outside Indonesia. However, identification of chemobrain for breast cancer survivor post-chemotherapy and the influencing factors remain an uncommon practice in Indonesia. This study is a part of a larger project about factors influencing chemobrain, but the scope in this study only emphasizes certain factors such as stress, age, duration of education, and sleep quality. The objective of this study was to identify the factors that are associated with verbal learning and memory function in post-chemotherapy breast cancer survivors by using the Indonesian version of the Hopkins Verbal Learning Test (HVLT).

## Methods

This study was a cross-sectional study conducted in 82 post-chemotherapy breast cancer survivors, 81 non-chemotherapy survivors, and 80 non-cancer female patients in Fatmawati General Hospital in Special Capital Region Jakarta and Hasan Sadikin hospital in Bandung, West Java. The subjects were recruited by using consecutive sampling. Inclusion criteria of post-chemotherapy survivor was have been treated ≥6 cycles of chemotherapy, and inclusion criteria of non-chemotherapy survivors was never treated with chemotherapy. Eligible criteria for all respondents were: (1) in age range 20–55 years, (2) be able to read and write, and (3) have no history of psychiatric disorders, any neurological disease and brain cancer. The sample size was based on thumb formula. In multivariate research, the sample size should have a number of times larger (preferably 10 times or more) than the number of variables in research (Dahlan, 2016).

Sample size in this study was calculated by formula for multivariate predictive analysis by Dahlan (2016):

*N* = (10*V)/P

*N* = sample size

*V* = number or independent variable

*P* = incidence of cognitive impairment after chemotherapy

This study consisted of 10 independent variables. Incidence of cognitive impairment after chemotherapy was about 41%. It was calculated by result review of Pendergrass, Targum, and Harrison (2018); it has been estimated that 13 to 70 percent of patients receiving cancer chemotherapy have measurable cognitive impairment. The mean value of incidence is 41%. In advance, sample size in this study is (10 × 10)/0.41 = 243.

### Measurements

The forms were used to collect information on the age, duration of education, marital status, survivor classification, chemotherapy type, menopausal status, recent hemoglobin (Hb) levels, and hormonal therapy usage. Data on verbal learning and memory function were collected by using the Indonesian version of the HVLT (a questionnaire). This version was developed by Hogervorst et al. (2011). The test consisted of recalling 12 specific words and was repeated three times. The questionnaire was scored from 0 to 36, with lower scores indicating greater cognitive decline. The questionnaire’s sensitivity is 87% and specificity is 98% at the cutoff point of 14.5. The scores for probable dementia were <14.5 (Hogervorst et al., 2011).

Data on quality of sleep was collected by using the Pittsburgh Sleep Quality Index (PSQI), which had been tested for validity and reliability in Indonesia. The research of Alim (2015) in Jakarta on the PSQI Indonesian edition obtained a Cronbach’s alpha value for internal consistency of 0.79, a content validity of 0.89, and a construction validity result that showed good component correlation with the PSQI global score and known group validity (*p* < 0.001). This study also obtained a sensitivity value of 1, precision of 0.81 for sensitivity, and a cutoff value of 5.

Stress was measured by using the Perceived Stress Scale 10 (PSS-10) instrument, which consisted of 10 questions with scores ranging from 0 to 40 (higher scores indicated greater stress). Fatigue was measured by using the Piper Fatigue Scale 12 (PFS-12) instrument, which consisted of 12 questions with scores ranging from 0 to 10. The stress and fatigue instruments were translated into Indonesian by researchers and then retranslated back into English after consultation with nursing experts. The Cronbach’s alpha values were 0.81 for PSS-10 and 0.84 for PFS-12.

### Collecting Data Method

The entire study was conducted only after obtaining ethical approval from the Research Ethics Committee of the Faculty of Nursing of Universitas Indonesia (Number 250/UN2.F12.D/HKP.02.04/2017) and after permission had been granted by local authorities. All respondents were provided clear information about the research and signed the informed consent form before participating in the study. The study began with asking demographic data, validating data on patient report, testing verbal learning and memory function using HVLT, and then requesting respondents to fill out the questionnaires. Data collection on post-chemotherapy patients was conducted after the patients had passed 6 cycles of chemotherapy.

### Statistical Analysis

Descriptive statistics were used to describe the characteristics of the respondents and the distribution of all variables. The bivariate associations between the pairs of all variables were tested by using the chi-squared and *t*-tests. Logistic multiple regression analysis was used to identify the associations of the variables with verbal learning and memory function. Data analyses were conducted by using SPSS version 20 statistical software. Two-sided significance tests were used (alpha < 0.05).

## Results

### Respondents

The mean age of the respondents was 43.06 (8.18) years, 196 (80.7%) of the respondents’ had been educated for <12 years, 82 (33.7%) were post-chemotherapy survivors, 46 (18.9%) were using hormone therapy, and 125 (51.4%) had gone through menopause. Among the remaining respondents, 91 (37.4%) were anemic, 124 (51.0%) had good sleep quality, and 115 (47.32%) experienced moderate fatigue. See Table 1.

**Table 1:** Respondent Characteristics (n = 243)

		Verbal learning and memory			
	Total	Possible dementia	Normal		*P*-value
Age, years [mean (SD)]	43.06 (8.18)	46.03 (7.57)	42.59 (8.19)		0.025
Stress [mean (SD)]	13.12 (5.55)	17.12 (4.98)	11.82 (5.26)		<0.001
Duration of education [n (%)]					
<12 years	197 (81.1)	30 (15.2)	167 (84.8)		0.189
≥12 years	46 (18.9)	3 (6.5)	43 (93.5)		
Survivor categories					
Post-chemotherapy	82 (33.8)	21 (25.6)	61 (74.4)		<0.001
Non-chemotherapy	81 (33.3)	8 (9.9)	73 (90.1)		
Non-cancer	80 (32.9)	4 (5.0)	76 (95.0)		
Chemotherapy categories					
First line	47 (19.3)	11 (23.4)	36 (76.6)		0.001
Second line	27 (11.1)	7 (25.9)	20 (74.1)		
Third line	8 (3.3)	3 (37.5)	5 (62.5)		
Non-chemotherapy	161 (66.3)	12 (7.5)	149 (92.5)		
Hormone therapy usage					
Yes	46 (18.9)	8 (17.4)	38 (82.6)		0.549
No	197 (81.1)	25 (12.7)	172 (87.3)		
Menopause status					
Yes	125 (51.4)	20 (16.0)	105 (84.0)		0.344
No	118 (48.6)	13 (11.0)	105 (89.0)		
Hemoglobin level					
Anemic	91 (37.4)	13 (14.3)	78 (85.7)		0.956
Non-anemic	152 (62.6)	20 (13.2)	132 (86.8)		
Quality of sleep					
Poor	124 (51.0)	30 (24.2)	94 (75.8)		<0.001
Good	119 (49.0)	3 (2.5)	116 (97.5)		
Fatigue					
Severe	7 (2.9)	1 (14.3)	6 (85.7)		0.026
Moderate	111 (45.7)	23 (20.7)	88 (79.3)		
Mild	115 (47.3)	8 (7.0)	107 (93.0)		
None	10 (4.1)	1 (10.0)	9 (90.0)		
Verbal learning and memory function		33 (13.6)	210 (86.4)		

### Prevalence of Verbal Learning and Memory Function

Thirty-three (13.6%) of the respondents were assessed as probably having dementia, and 21 (25.6%) of the post-chemotherapy respondents were assessed as probably having dementia. See Table 1.

### Factors Affecting Verbal Learning and Memory Function

The most dominant factor associated with verbal learning and memory function in this study was stress, which has an odds ratio (OR) score of 1.159; for the highest individual stress level, the risk of dementia was 1.159 times higher than the average stress level. The study factors of education, sleep quality, age, and stress explained approximately 34.2% of the differences in verbal learning and memory (probable dementia). This result is shown in Table 2.

**Table 2:** A Logistic Regression Analysis of Factors Affecting Verbal Learning and Memory Function (n = 243)

Variable	*B*	*P*-value	OR	95% CI	*R*^2^
Education length	−1.047	0.127	0.351	0.091–1.347	0.342
Sleep quality	−2.257	0.000	0.105	0.030–0.366	
Age	0.052	0.070	1.054	0.996–1.115	
Stress	0.148	0.000	1.159	1.073–1.252	
Constant	-5.539				

## Discussion

Among all study respondents, 21 (25.6%) of the post-chemotherapy survivors had probable dementia. Data of cognitive deficit varies on patients who get chemotherapy treatment (Jim et al., 2012). This percentage was similar to that of other studies that found that 15%–39% of cancer survivors had experienced cognitive problems within months to years after treatment (Ahles, Root, & Ryan, 2012; Janelsins et al., 2011; Oh, 2017), and 20% of those subjects had been objectively confirmed with decreased cognitive function (Oh, 2017). These data confirmed that the primary concern of breast cancer survivors was cognitive dysfunction, which is commonly known as chemobrain or chemo-fog. This phenomenon has become a research focus in cancer survivors by health workers (Selamat et al., 2014). This condition greatly affects the lives of survivors by causing frustration and discouragement and by negatively influencing their confidence and social relationships, thus possibly causing difficulties in implementing and adapting compensation strategies (Boykoff et al., 2009; Von Ah et al., 2013). Post-chemotherapy survivors can experience memory loss, decision-making difficulties, and impaired speech. Furthermore, married women have reported frustration because their household role was diminished (Cheung et al., 2012). To help manage this condition, adequate assessment and intervention by health care providers are needed in this population.

The data is different from other research, which shows 19% of respondents experiencing cognitive decline after chemotherapy, specifically delayed memory (Andryszak, Wiłkość, Żurawski, & Izdebski, 2018). This may have occurred because the respondents only get four cycles of chemotherapy, with effects being milder. The other research also shows 16.9% of respondents experiencing significant cognitive decline after chemotherapy, specifically in verbal learning and memory function (Root, Andreotti, Tsu, Ellmore, & Ahles, 2016). This may happen because the respondents only get 3–5 cycles of chemotherapy. However, in our research, the respondents passed six cycles of chemotherapy.

In this study, the other significant factors were education length, sleep quality, and age. These four variables affected the incidence of decreased verbal learning and memory function by as much as 34.2%. Similar to previous research, hierarchical regression analysis showed that age, sex, fatigue, and depression explained 49.6% of the variations in self-reported cognitive decline (Moon, Kim, & Kim, 2011). Logistic regression analysis showed that stress had the strongest effect (OR 1.159) on decreasing verbal learning and memory function. This finding was similar to those of other studies in which mood disorders were found to be factors in cognitive impairment in post-chemotherapy breast cancer survivors (Moon et al., 2011).

The mean stress score was 17.12 (4.98) in probable dementia respondents and was 14.62 (4.53) in post-chemotherapy respondents (scores of 14–26 indicate moderate stress). These results were different from those of the infertile female population in Surabaya, Indonesia, which showed that 40% of the respondents had mild stress and that 56.7% of the respondents had moderate stress (Setiyono, Hendarto, Prasetyo, & Maramis, 2015). This difference might be because cancer is potentially one of the most stressful illnesses, and chemotherapy treatments have often caused distress in cancer patients (LeMone, Burke, Bauldoff, & Gubrud-Howe, 2015).

In another study, stress was found to significantly affect verbal learning and memory function and was one of the many causative factors that affected cognitive impairment in survivors after receiving chemotherapy (Hermelink et al., 2017). Psychological and emotional stress may interfere with the pituitary–adrenal axis of the hypothalamus and the sympathetic nervous system and disrupts the immune system (Irwin & Cole, 2011). The mechanism of this impairment is realized via the development of cytokine proinflammation. When cytokines reach brain tissue, immune cells, such as microglia, trigger the production of other cytokine proinflammation and inflammation mediators. Cytokine proinflammation increases the metabolism of the key neurotransmitters, namely, noradrenaline, dopamine, and serotonin. Animal studies have shown that proinflammation circulation may alter the learning process and memory in animals. Cytokines can alter long-term potentiation in the hippocampus and interfere with memory consolidation (Asher & Myers, 2015).

This research also found that age significantly affects verbal learning and memory. This is in line with other studies which found that age can affect cognitive function after chemotherapy (Dietrich, Monje, Wefel, & Meyers, 2008). According to Hess and Insel (2007), age is one of the moderator factors in post-chemotherapy cognitive impairment. In this study, the accumulating age was related to cognitive decline. This study limited the age of respondents to 55 years old, yet the cognitive decline still was evident as a side effect of chemotherapy.

Education duration was included into the logistic regression equation. Results were similar to another study, which found a positive relation between education duration with verbal function and memory, with the average duration of respondents’ education of 13.9 years (Andryszak et al., 2018). Meanwhile, education is a moderating factor in post-chemotherapy cognitive impairment (Hess & Insel, 2007). The majority of respondents in this study had an educational duration of ≤12 years. Another study of breast cancer patients in Indonesia also found that respondents who had education ≤12 years were 94.7% (Fitri, Maneewat, & Sangchan, 2017). It may be due to the fact that Indonesia is one of the developing countries.

In this study, sleep quality also significantly affected verbal learning and memory functions. This was similar to another study that found a significant positive relationship between working memory capacity and increased memory appearance after sleep (Fenn & Hambrick, 2012). However, the mechanism that explains this relationship cannot be fully explained (Peigneux & Smith, 2011). Further research is needed to identify this relationship.

### Study Limitations

This study only identifies the factors as previously described, but not several other factors that might affect the incidence of cognitive impairment in breast cancer survivor post-chemotherapy. These factors include diet, exercise, social support, and genetic factors (Asher & Myers, 2015; Hess & Insel, 2007; Myers, 2009). For further study, it is better to identify all of these factors.

### Implication for Future Research and Practice

Assessment of cognitive function in breast cancer survivor post-chemotherapy is essential. Nurses have to always improve their knowledge and skills about cognitive function in breast cancer survivor post-chemotherapy, to be able to improve the quality of nursing care in this population. Training and research on the phenomena can assist the nurses to improve the quality of care.

## Conclusion

The study results showed that age, stress, survivor type, chemotherapy type, sleep quality, and fatigue were significantly associated with verbal learning and memory function. Stress was the most dominant factor associated with verbal learning and memory function. Therefore, it is important to consider these factors in the assessment and care management of post-chemotherapy breast cancer survivors. Other interventions should be studied to reduce the risks of chemotherapy-induced cognitive impairment as much as possible.

## Declaration of Conflicting Interests

The authors declared no potential conflicts of interest with respect to the research, authorship, and/or publication of this article.

## Funding

This work was supported by Beasiswa Program Pasca-Sarjana Dalam Negeri (BPPDN), the Ministry of Research, Technology and Higher Education of the Republic of Indonesia, and Hibah Tugas Akhir Doktor of Universitas Indonesia (1252/UN.R3.1/HKP.05.00/2018).
